# Elevated Serum Level of IL-35 Associated with the Maintenance of Maternal-Fetal Immune Tolerance in Normal Pregnancy

**DOI:** 10.1371/journal.pone.0128219

**Published:** 2015-06-04

**Authors:** Chao-yan Yue, Bin Zhang, Chun-mei Ying

**Affiliations:** Department of Laboratory Medicine, Obstetrics and Gynecology Hospital of Fudan University, Shanghai, China; Cincinnati Children's Hospital, UNITED STATES

## Abstract

**Objectives:**

IL-35 is a novel inhibitory cytokine. In this study, we investigate the serum levels of inhibitory cytokines IL-35, IL-10 and TGF-β in both normal pregnancies and non-pregnant females, and whether IL-35 is associated with the pathogenesis of recurrent spontaneous abortion. We also try to elucidate the relationships of IL-35 with estrogen and alpha-fetoprotein (AFP).

**Methods:**

The levels of IL-35, IL-10, TGF-β, estradiol (E2), unconjugated estriol (uE3) and AFP were analyzed in 120 normal pregnancies, 40 women suffering recurrent spontaneous abortion, 40 postpartum healthy women and 40 non-pregnant women by enzyme-linked immunosorbent assay (ELISA). The correlations between inhibitory cytokines, estrogen and AFP were assessed with the Spearman rank correlation coefficient.

**Results:**

Data are expressed as median and percentiles (Q1, Q3).The level of serum IL-35 in normal pregnancies was significantly higher than that in non-pregnant women [333.6 (59.32, 1391) pg/mL *vs*. 123.9 (8.763, 471.7) pg/mL; *P *< 0.001]. A significantly higher level of TGF-β was observed in the first trimester only as compared to non-pregnant women [473.4 (398.0, 580.5) pg/mL *vs*. 379.7 (311.0, 441.3) pg/mL, *P* < 0.01]. The difference in serum IL-10 level between pregnant women and non-pregnant women was not significant [8.602 (5.854, 12.89) pg/mL *vs*. 9.339 (5.691, 12.07) pg/mL; *P* > 0.05]. The level of serum IL-35 in recurrent spontaneous abortion was significantly lower than that in normal early pregnancy [220.4 (4.951, 702.0) pg/mL *vs*. 386.5 (64.37, 1355) pg/mL; *P *< 0.05]. The higher IL-35 level in first trimester pregnant women correlated with E2 (r = 0.3062, *P* < 0.01) and AFP (r = 0.3179, *P* < 0.01).

**Conclusion:**

Serum levels of IL-35 increased in normal pregnancy and decreased in recurrent spontaneous abortion. Increased IL-35 correlated with estrogen and AFP levels in early pregnancy. IL-35 is becoming recognized as an active player in the maintenance of a successful pregnancy, but this is not the case for IL-10 or TGF-β.

## Introduction

Pregnancy is a major challenge for the maternal immune system. The foreign antigens expressed by the fetus can even stimulate the immune system. In this complex immunological dilemma, the maternal immune system actively responds to fetal antigens with the help of endocrine pathways [1,2].

Regulatory T cells (Treg) play an important role in maintaining immune tolerance, inhibiting progression of autoimmune disease and preventing excessive inflammatory response [3]. Additionally, a specific role in the maintenance of fetal immune tolerance has been widely reported for these cells in both humans and mice [4–15].

Cytokine pathways are considered to be the major mechanism for immunosuppression of Tregs. Inhibitory cytokines include IL-10, TGF-β and IL-35. IL-35 is an inhibitory cytokine first identified in 2007, which is produced primarily by CD4^+^Foxp3^+^Treg cells and required for the suppressive activity of regulatory T-cell populations [16–18]. IL-35 is also produced by activated B cells, tolerogenic dendritic cells and to a lesser extent by activated endothelial cells, smooth muscle cells, and monocytes [19–23].

Although IL-10, TGF-β and IL-35 are all inhibitory, the extent of their suppression, and of their non-overlapping functions, needs further clarification [24,25]. There is a general consensus that Tregs increase in decidual tissue, peripheral blood and lymphoid organs during pregnancy and mediate maternal tolerance to the fetus [4–15]. However, information on the level of serum IL-35 and its potential role in pregnancy remains limited. In this study, we examined the serum levels of inhibitory cytokines in normal pregnancy and recurrent spontaneous abortion, and analyzed the correlation of IL-35 with estrogen and alpha-fetoprotein (AFP) levels.

## Materials and Methods

### Subjects

A total of 120 normal pregnancies, 40 women subject to recurrent spontaneous abortion, 40 postpartum healthy women and 40 non-pregnant women from the Obstetrics & Gynecology Hospital of Fudan University, Shanghai, China, were enrolled in this study between October 2013 and December 2014. Normal pregnancies were divided into three groups: the first trimester covers weeks 0 through 12; the second trimester covers weeks 13 through 27; the third trimester covers weeks 28 through the birth of the baby. Clinical diagnosis determined that the cause of women undergoing recurrent spontaneous abortion was not due to anatomical, endocrine, or genetic factors. Blood was collected from the recurrent spontaneous abortion women before the abortion happened, during the period of threatened abortion. Forty age-matched, healthy, postpartum female donors and forty age-matched, healthy, non-pregnant female donors (NP) served as controls. None of the participants suffered from autoimmune or inflammatory diseases, or were taking steroid hormones or antibiotics before samples were collected. Before sample collection, approval was obtained from the Research Ethics Committee of the Obstetrics & Gynecology Hospital of Fudan University, and written consent was obtained from all women in this study.

### Serum samples

Five milliliters of peripheral blood were collected from participants. The peripheral blood was collected in a serum separator tube and samples were allowed to clot for 30 min before centrifugation at 1000 × *g* for 15 min. All peripheral blood samples were processed within 2 h of collection. Serum was removed and assayed immediately or aliquoted and stored at -20°C or -80°C until analysis. Samples were only thawed once.

### ELISA detection of the levels of serum cytokines

The serum levels of IL-35, IL-10 and TGF-β were detected quantitatively with enzyme-linked immunosorbent assay (ELISA) kits (Westtang Bio-tech, Shanghai, China), used according to the manufacturer’s instructions. The detection limits of IL-35, IL-10 and TGF-β were 15 pg/mL, 1 pg/mL and 15 pg/mL, respectively. Both intra-assay and inter-assay coefficients of variation were <10%. Each sample was run in duplicate and the mean value was used.

### Measurement of serum estrogen and AFP

Serum samples were analyzed for estradiol (E2), unconjugated estriol (uE3) and AFP using Beckman Coulter instruments. The analytical performance of the measurements assessed with control materials showed values within the recommended limits. E2 was measured by a competitive enzyme immunoassay using the manufacturer’s reagents. This assay has a functional sensitivity of 20 pg/mL, an analytical reporting range of 20 to 4800 pg/mL, and the coefficient of variation was <12%. uE3 was measured on the same instrument with a functional sensitivity of 0.017 ng/mL, an analytical reporting range of 0.017 to 6.9 ng/mL, and the coefficient of variation was <10%. AFP was measured by a two-site (sandwich) enzyme immunoassay using the manufacturer’s reagents. The functional sensitivity of this assay was 0.50 ng/mL, the analytical reporting range was 0.5 to 3000 ng/ml, and the coefficient of variation was <8%. Each sample was run in duplicate and the mean value was used.

### Statistical analysis

Data are expressed as median and percentiles (Q1, Q3). For data with normal distribution and homogeneity of variance, an independent-sample *t* test was adopted to compare differences between two groups, and one-way ANOVA with Tukey’s post-hoc test was performed if there were three or more means. For non-normally distributed data, differences between groups were evaluated by the non-parametric Mann-Whitney U test, and association analysis was assessed with a Spearman rank correlation coefficient. All statistical analyses were performed by means of GraphPad Prism version 5.0 for Windows (GraphPad Software, USA) and *P* < 0.05 was considered a significant difference.

## Results

**Fig 1:**
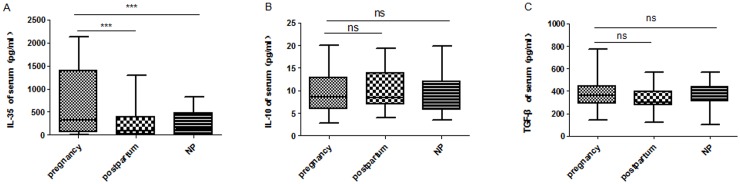
The serum levels of inhibitory cytokines (A) IL35, (B) IL10, and (C) TGF-β in pregnancy. These data show medians and percentiles over all patients throughout their pregnancies. NP: non-pregnant women; postpartum: postpartum healthy women; *** *P* < 0.001; ns: not significant.

Serum levels of IL-35, IL-10 and TGF-β were detected in all participants. Data are expressed as median and percentiles (Q1, Q3). Fig 1 shows that the level of serum IL-35 in normal pregnancies was significantly higher than that in non-pregnant women [333.6 (59.32, 1391) pg/mL *vs*. 123.9 (8.763, 471.7) pg/mL; *P* < 0.001]. The differences in serum IL-10 [8.602 (5.854, 12.89) pg/mL *vs*. 9.339 (5.691, 12.07) pg/mL; *P* > 0.05] and TGF-β [364.4 (291.0, 448.4) pg/mL *vs*. 379.7 (311.0, 441.3) pg/mL; *P* > 0.05] levels between pregnant women and non-pregnant women were not significant.

**Fig 2:**
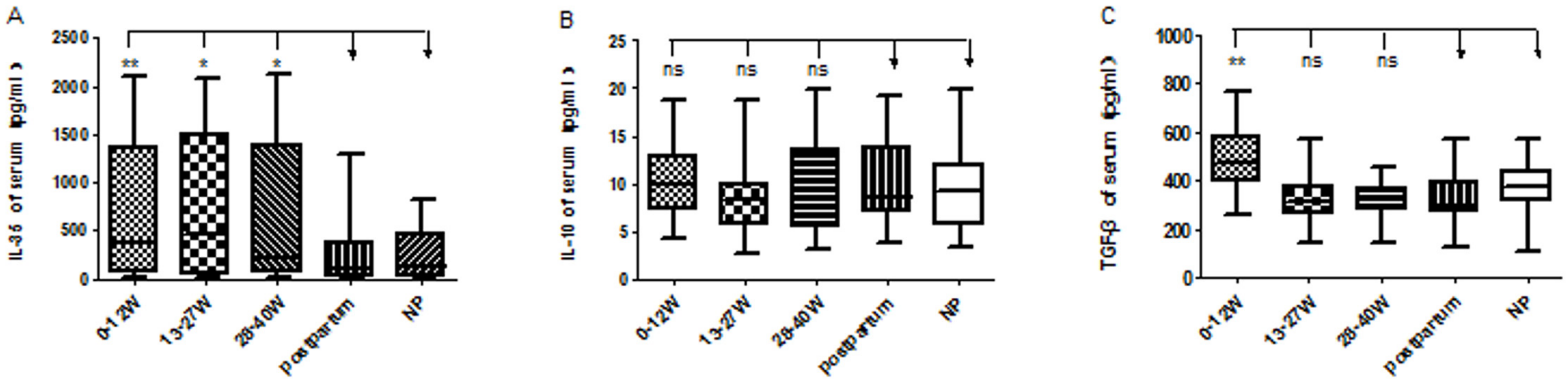
The serum levels of inhibitory cytokines (A) IL35, (B) IL10, and (C) TGF-β at different stages of pregnancy. 0–12 weeks: first trimester; 13–27 weeks: second trimester; 28–40 weeks: third trimester. NP: non-pregnant women; postpartum: postpartum healthy women; *P < 0.05; ** *P* < 0.01; ns: not significant.

Fig 2 shoes cytokine levels throughout the pregnancy cycle. The levels of serum IL-35 were significantly increased in all stages of pregnancy [first trimester 386.5 (64.37, 1355) pg/mL, second trimester 460.4 (45.38, 1508) pg/mL, third trimester 211.9 (63.3, 1393) pg/mL] compared to the control groups [non-pregnant 123.9 (8.763, 471.7) pg/mL (*P* < 0.001), postpartum 95.74 (5.568, 385.9) pg/mL (*P* < 0.001)]. The difference in serum IL-10 level between pregnant women and controls was not significant [first trimester 9.860 (7.258, 12.87) pg/mL, second trimester 8.404 (5.570, 9.948) pg/mL, third trimester 8.456 (5.446, 13.68) pg/mL *vs*. non-pregnant 9.339 (5.691, 12.07) pg/mL (*P* > 0.05) and postpartum 8.514 (6.971, 13.84) pg/mL (*P* > 0.05)]. The levels of serum TGF-β were found to be increased in the early stages of pregnancy [first trimester 473.4 (398.0, 580.5) pg/mL, second trimester 310.4 (258.4, 379.6) pg/mL, third trimester 325.1 (279.4, 371.5) pg/mL] compared to the control groups [non-pregnant 379.7 (311.0, 441.3) pg/mL, postpartum 298.9 (271.4, 394.9) pg/mL]. Thus, TGF-β was significantly increased in the first trimester of pregnancy (*P* < 0.01), but the differences were not significant in the second and third trimesters (*P* > 0.05).

**Fig 3:**
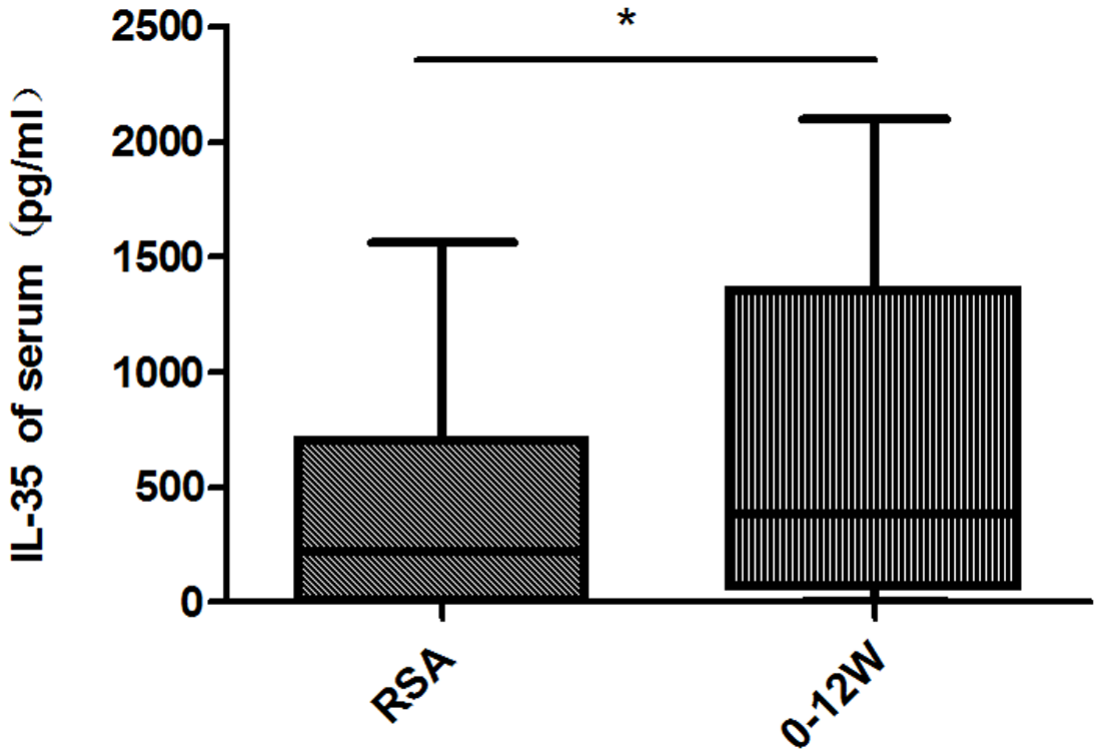
The serum levels of IL-35 in women suffering recurrent spontaneous abortion. 0–12 weeks: first trimester; RSA: recurrent spontaneous abortion; * *P* < 0.05; ns: not significant.

Cytokine levels in women suffering recurrent spontaneous abortion are shown in Fig 3. The level of serum IL-35 was significantly lower than that in normal early pregnancy [220.4 (4.951, 702.0) pg/mL *vs*. 386.5 (64.37, 1355) pg/mL; *P* < 0.05].

The immunological interaction between fetus and mother is a paradoxical communication, which is regulated by fetal antigen presentation and by the recognition and reaction of the maternal immune system to these antigens [26]. AFP is one of the oncofetal antigens produced during fetal development. During pregnancy, oncofetal antigens, produced in considerable concentrations during fetal growth and differentiation, affect the maternal immune response and generate maternal tolerance toward the embryo, in the manner of host defense in carcinogenesis [27]. Sex hormones such as human chorionic gonadotropin, estrogens, progesterone, and others contribute to induction of immunologic tolerance at the beginning of gestation [28]. Estrogen can influence the development, maturation and function of the female reproductive tract. There are three major estrogen hormones: estrone, estradiol (E2), and estriol (E3). E2 is the most important estrogen in non-pregnant females; E3 becomes the primary form of estrogen in the body during pregnancy.

**Fig 4:**
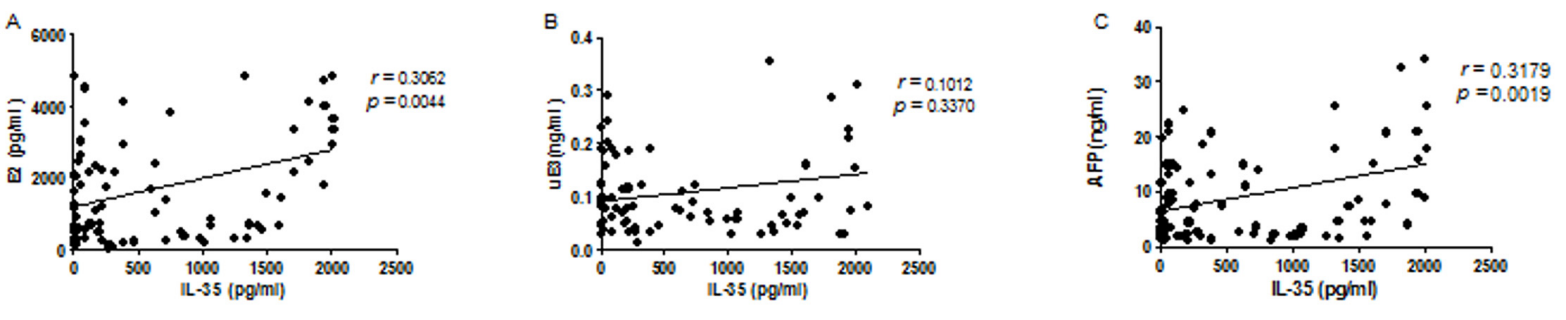
The correlation of estrogen and AFP levels with IL-35 in early pregnancy. *P* < 0.01 represents a significant correlation using the Spearman method.

The consistency of increased serum estrogen, AFP and serum IL-35 levels in pregnancies encouraged us to perform a correlation study. Our results showed that in the first trimester, increased IL-35 was positively correlated with E2 (*r* = 0.3062, *P* < 0.01) and AFP (*r* = 0.3179, *P* < 0.01). Increased IL-35 was correlated with uE3, but the correlations were not significant (*P* > 0.05) (Fig 4).

## Discussion

The immunological relationship between mother and fetus is still mysterious. Many of the parameters involved in the maternal-fetal interaction have been clarified by molecular immunology, such as that the maternal immune system is tightly regulated by hormone release and cytokine action to protect the developing fetus [26, 28].

In this study, we investigated serum IL-35 levels in normal pregnancy and women suffering recurrent spontaneous abortion compared to non-pregnant women and postpartum healthy women. We also assessed the correlation of the IL-35 level with estrogen and AFP in early pregnancy. We demonstrated that inhibitory cytokines IL-35, IL-10 and TGF-β were elevated to different extents during pregnancy, and IL-35 plays an important role in maternal-fetal immune tolerance. In the first trimester of pregnancy, the increased immunosuppressive factors were mainly IL-35 and TGF-β; in the second and third trimesters of pregnancy the main immunosuppressive factor was IL-35. During recurrent spontaneous abortion, IL-35 decreased significantly. In early pregnancy, the IL-35 level positively correlates with E2 and AFP levels. This may be due to estrogen and AFP regulated proliferation of Tregs, which indirectly influenced the secretion of inhibitory cytokines. To our knowledge, this is the first report to show the correlation of IL-35 level with increased estrogen and AFP in pregnancy.

Tregs are essential for the maintenance of immune tolerance. In early pregnancy, maternal tolerance is important to allow invasion of fetal trophoblasts to anchor the placenta. Previous work showed that during early pregnancy, expansion of the Treg pool normally takes place; a decrease in the amount and suppressive capacity of Tregs is accompanied by recurrent spontaneous abortions [1, 8, 11, 15, 29–32]. However, the mechanisms of Tregs mediating suppression remain controversial. In this study, we first found that the level of inhibitory cytokine IL-35 was significantly higher in normal pregnancy than in age-matched non-pregnant female donors. Our results indicate that increased IL-35 in normal pregnancy may provide immune protection for the fetus, and insufficient IL-35 is involved in the recurrent spontaneous abortion pathogenesis, so IL-35 associated with the establishment and maintenance of maternal-fetal tolerance during a successful pregnancy. It has been reported that first-trimester human trophoblast cells expressed and secreted IL-35, which might be of benefit to the suppressive capacity of maternal immune cells [33]. In mouse models, IL-35 positive cells in the uterus showed significant differences in distribution after fetal implantation; they were mainly distributed in the luminal epithelium and glandular epithelium of the mouse uterus from gestational day 1 to 2, and in the glandular epithelium and stroma from gestational day 4 to 7 [34]. In women with history of idiopathic recurrent pregnancy loss, IL-35 was found to be significantly lower compared to fertile controls [35]. Reduced IL-35 production in preeclamptic women may lead to lower cytokine inhibitory activity, which may account for the increased proteinuria and blood pressure [36]. IL-35 as a recently identified member of the IL-12 family of cytokines offers potential as a target for new therapies for autoimmune, inflammatory, and infectious diseases [22,23].

Some studies have found that levels of inhibitory cytokines IL-10 and TGF-β increased during pregnancy [37]. In our work, we found that TGF-β significantly increased in the first trimester only. Previous work from Clark and colleagues suggested that vaginal TGF-β promotes a regulatory T-cell response enhancing the success of pregnancy [38]. Moreover, by depleting Tregs in different pregnancy stages, Shima and colleagues showed that Tregs are important for the implantation phase and early stage of pregnancy, but might not be necessary for maintenance of the late pregnancy stages [39]. This may explain why we observed no significant change in TGF-β levels relative to non-pregnant women during the latter stages of pregnancy.

In our work, we found the difference in serum IL-10 level between pregnant women and controls was not significant, which is consistent with the previous study of Svensson et al. They point out that IL-10 and IL-4 deficient mice have normal pregnancies and neither of these cytokines is crucial for fetal survival [40].

Our results are consistent with the conclusion that human Tregs express and require IL-35 for maximal suppressive capacity, as proposed by Collision et al. They found substantial up-regulation of EBI3 and IL-12A, but not IL-10 or TGF-β, in activated Tregs compared with conventional human T cells. Contact-independent Treg-mediated suppression was IL-35 dependent but did not require IL-10 or TGF-β. Human Treg-mediated suppression led to the conversion of Tconvs into iTr35 cells (an IL-35-induced Treg population), in an IL-35-dependent manner. Thus, IL-35 contributes to human Treg-mediated suppression, and its conversion of Tconv into iTr35 cells may contribute to maximal suppressive ability [41]. Humoral immunity has also been shown to be suppressed by rIL-35, when it induced the conversion of human B cells into regulatory B cells that produce IL-35 as well as IL-10 [19].

Hormonal changes during pregnancy have been shown to influence the generation, expansion and suppressive capacity of Tregs. Hormonal modulation of the induction of adaptive and constitutive Tregs appears to underlie and/or be one of the mechanisms of peripheral tolerance to the fetus during pregnancy. AFP is one of the oncofetal antigens with intrinsic immunoregulatory properties [42,43]. Our results showed that the serum concentration of IL-35 was positively correlated with E2 and AFP in early pregnancy. Correlation does not prove causation, but previous studies provide interesting clues to better understand the mechanisms of immunoregulation by estrogen and AFP. Shirshey and colleagues showed that estrogen, progesterone and HCG increase Treg levels in PBMCs during pregnancy compared to non-pregnant women [43]. Furthermore, estradiol at the doses found in pregnancy could induce expansion of Tregs, enhance Treg function, and induce the phenotype of Tregs in activated responder T cells, in both humans and mice [44–49]. Tai et al. found that E2 at physiological doses stimulated the conversion of CD4^+^CD25^-^T cells into CD4^+^CD25^+^T cells in vitro. They also found that the estrogen receptor (ER) exists in CD4^+^CD25^-^T cells and E2 may directly act on CD4^+^CD25^-^T cells via ER(s) [45]. Alisa et al. demonstrated that AFP may contain specific epitopes which activate the expansion of inducible TGF-β producing regulatory T cells, leading to evasion of tumor control [42]. Given that estrogen and AFP may contribute to the development of Tregs, we suggest that estrogen and AFP may also contribute to the expression of inhibitory cytokines produced by Tregs; our work supports this possibility. The increased estrogen and AFP levels are not the direct reason for the elevation of IL-35. Further studies are necessary to elucidate the mechanisms of IL-35 function in reproduction.

IL-35 as a potent anti-inflammatory cytokine, which has been reported required for the suppressive activity of regulatory T-cell populations [16–18]. But the latest research proved that Treg are not the only source of IL-35, regulatory B cells and tolerogenic dendritic cells also can produce IL-35[19–21]. Cellular source of IL-35 was not determined is the limitations of our study, further studies are necessary to elucidate cellular source of IL-35 in the maternal-fetal immune tolerance by intracellular cytokine staining. The cellular source of IL-35 is uncertainly, however there is a general consensus that IL-35 is required for optimal suppression of immune responses. IL-35 suppresses the immune response by up-regulating regulatory T cells, inducing the generation of the anti-inflammatory milieu via new Treg populations (iTreg, iTr1, and iTr35 cells) or IL-35-producing B cells, and by inducing anti-inflammatory effects through inhibition of both Th1 and Th17 responses. The biological activities of IL-35 are improving our expectations in therapies and strategies for intractable immune diseases [50–52].

Inhibitory cytokines as well as endocrine pathways and antigen-dependent mechanism seem to contribute to Treg augmentation and functionality during pregnancy. The fundamental mechanisms of IL-35 function in reproduction are not entirely clear, but both antigens and a hormonal input seem associated with the increase in IL-35 level. The persistence of IL-35 during pregnancy indicates a potential and promising option in the treatment of infertility, miscarriage, pregnancy complications, and in assisted reproductive techniques.

## Conclusion

We confirmed that the serum level of IL-35 is elevated in normal pregnancy and correlates with increased estrogen and AFP in early pregnancy. The IL-35 level is decreased in recurrent spontaneous abortion. We provide convincing data that IL-35 is an active player in the maintenance of a successful pregnancy, but this is not the case for IL-10 or TGF-β. Our data support the application of IL-35 in the improvement of fertility therapy.
